# Characterization of human respiratory syncytial virus (RSV) isolated from HIV‐exposed‐uninfected and HIV‐unexposed infants in South Africa during 2015‐2017

**DOI:** 10.1111/irv.12727

**Published:** 2020-03-03

**Authors:** Hui Liu, Bin Lu, David E. Tabor, Andrey Tovchigrechko, Deidre Wilkins, Hong Jin, Shabir A. Madhi, Nasiha Soofie, Mark T. Esser, Marta C. Nunes

**Affiliations:** ^1^ AstraZeneca South San Francisco CA USA; ^2^ AstraZeneca Gaithersburg MD USA; ^3^ Department of Science and Technology/National Research Foundation: Vaccine Preventable Diseases Faculty of Health Sciences University of the Witwatersrand Johannesburg South Africa; ^4^ Medical Research Council: Respiratory and Meningeal Pathogens Research Unit Faculty of Health Sciences University of the Witwatersrand Johannesburg South Africa

**Keywords:** antigenic sites, G and F proteins, monoclonal antibodies, RSV

## Abstract

**Background:**

RSV is a leading cause of lower respiratory tract infection in infants. Monitoring RSV glycoprotein sequences is critical for understanding RSV epidemiology and viral antigenicity in the effort to develop anti‐RSV prophylactics and therapeutics.

**Objectives:**

The objective is to characterize the circulating RSV strains collected from infants in South Africa during 2015‐2017.

**Methods:**

A subset of 150 RSV‐positive samples obtained in South Africa from HIV‐unexposed and HIV‐exposed‐uninfected infants from 2015 to 2017, were selected for high‐throughput next‐generation sequencing of the RSV F and G glycoprotein genes. The RSV G and F sequences were analyzed by a bioinformatic pipeline and compared to the USA samples from the same three‐year period.

**Results:**

Both RSV A and RSV B co‐circulated in South Africa during 2015‐2017, with a shift from RSV A (58%‐61% in 2015‐2016) to RSV B (69%) in 2017. RSV A ON1 and RSV B BA9 genotypes emerged as the most prevalent genotypes in 2017. Variations at the F protein antigenic sites were observed for both RSV A and B strains, with dominant changes (L172Q/S173L) at antigenic site V observed in RSV B strains. RSV A and B F protein sequences from South Africa were very similar to the USA isolates except for a higher rate of RSV A NA1 and RSV B BA10 genotypes in South Africa.

**Conclusion:**

RSV G and F genes continue to evolve and exhibit both local and global circulation patterns in South Africa, supporting the need for continued national surveillance.

## 1 INTRODUCTION

Respiratory syncytial virus (RSV) is the most common cause of acute lower respiratory tract infection (LRTI) in children globally. It was estimated that in 2015, 33.1 million episodes of LRTI, 3.2 million hospitalizations, and as many as 118,200 deaths were attributable to RSV in children <5 years of age worldwide, with the greatest burden of RSV‐associated hospitalization and death occurring in infants younger than 6 months of age.^1^ Moreover, developing countries have a much higher incidence of severe RSV LRTI compared to developed countries, with approximately 91% of all RSV‐associated hospitalizations and 99% of deaths occurring in developing countries.^2^

The RSV genome encodes 11 proteins. The two surface glycoproteins, the fusion (F) and the attachment (G) protein, are crucial for virus infectivity and pathogenesis, and are the major antigens to stimulate the production of neutralizing antibodies.^3^, ^4^, ^5^ While the G protein is responsible for the attachment of the virus to the host epithelial cells, the F protein mediates viral entry by fusing viral and cellular membranes, leading to the subsequent release of viral RNA into the host cell cytoplasm.^6^ RSV has two subtypes, A and B, which are further characterized into different genotypes, based on antigenic and genetic variability of the second hypervariable region (HVR2) of the G protein.^7^ In contrast to the G protein, the F protein is well‐conserved between the two RSV subtypes and among different genotypes. Six consensus antigenic sites have been identified in the F protein, either in its pre‐fusion and/or post‐fusion conformation.^8^, ^9^, ^10^ Some of these sites are potential targets for prophylactic antibodies, such as site II, the target of the only approved anti‐RSV immunoprophylaxis for high‐risk infants (palivizumab); site Ø, the target of nirsevimab (MEDI8897)^11^; site IV, the target of MK‐1654^12^; and site V, the target of suptavumab.^13^, ^14^ Immune pressure, coupled with the RSV error‐prone RNA polymerase, leads to viral evolution and drift that should be carefully monitored.

Hospital surveillance studies from South Africa demonstrated that children born to HIV‐infected mothers, but not HIV‐infected themselves, have 1.4‐ to 2.1‐fold greater risk of RSV‐associated hospitalization and death, respectively, compared to HIV‐unexposed children.^15^, ^16^ The increased burden of RSV disease observed in HIV‐exposed infants is likely due to reduced transplacental transfer of RSV antibodies in women living with HIV.^17^ We investigated the molecular epidemiology and genetic variability of RSV isolates collected during 2015 to 2017 from HIV‐exposed‐uninfected and HIV‐unexposed hospitalized infants <12 months old in South Africa. The RSV sequence data obtained from South Africa were further compared to RSV sequences obtained from the USA during the same period.^18^

## 2 MATERIALS AND METHODS

### 2.1 Study population

Active surveillance was conducted at Chris Hani Baragwanath Academic Hospital (CHBAH) in Soweto, South Africa. Admissions to all pediatric medical wards, including a short stay ward, were screened for enrollment into the study from January 2015 to December 2017. The surveillance case definition included infants <3 months of age who were diagnosed with suspected sepsis or physician‐diagnosed LRTI, and infants 3‐12 months of age who had a physician‐diagnosed LRTI.

### 2.2 Ethics statement

The RSV surveillance study was approved by the Human Research Ethics Committee of the University of the Witwatersrand (131109) and conducted in accordance with the Good Clinical Practice guidelines. Caregivers were informed on the nature, purpose, and process of the study, and were provided written informed consent for the use of their infants’ samples in future studies on infectious diseases. Ethical approval for this specific analysis was also obtained from the same Ethics Committee (M170965).

### 2.3 Sample collection and testing

Nasopharyngeal swabs were collected using a commercially available nylon flocked tipped swab (FLOQS, Copan Flock Technologies) as previously described^19^ and were placed in 2.5 mL of universal transport media (UTM, Copan Flock Technologies). Samples were then transported on ice to the Respiratory and Meningeal Pathogens Research Unit (RMPRU) laboratories, on the premises of CHBAH. A multiplex real‐time polymerase chain reaction (PCR) assay was used to detect the presence of RSV. A cycle threshold (*C*_t_) value of <37 was used as a cutoff for identifying RSV‐positive samples.

**Figure 1:**
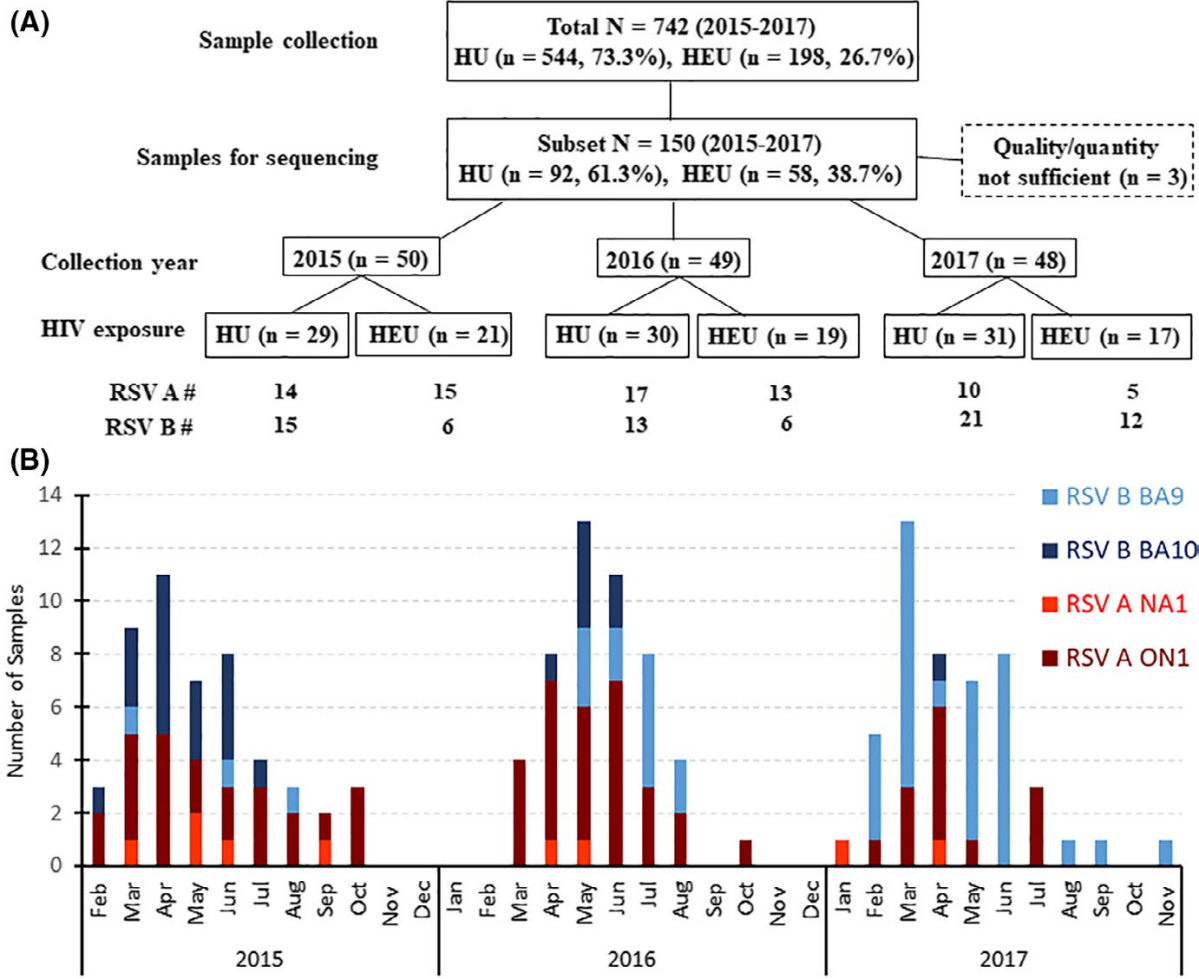
RSV‐positive sample consort diagram and distribution of RSV subtypes and genotypes by sample collection month over 2015 to 2017. A. Representative RSV‐positive (n = 150) samples from HIV‐unexposed (HU) (n = 544) and HIV‐exposed‐uninfected (HEU) infants (n = 198) from the 2015‐2017 RSV seasons in South Africa were selected for sequencing. The sample collection year, HIV exposure status, and RSV A or B subtype are shown. B. The subtypes and genotypes were determined from the 147 sequenced samples collected from January 2015 to December 2017

We selected 50 representative samples per year with *C*_t_ < 30 for sequencing analysis from a total of 742 samples obtained during 2015‐17. Samples were selected for HIV‐exposed‐uninfected (HEU) and HIV‐unexposed infants separately at a ratio of 2:3 and included at least one sample positive for each RSV subtype per week of each RSV season. A total of 150 samples with equal numbers of RSV A and B strains were transferred to AstraZeneca for sequencing. Sequence data from 147 samples were obtained and included in the analysis. A total of three samples were excluded from data analysis due to “quality/quantity not sufficient” (QNS). The sample consort diagram is shown in Figure 1A.

### 2.4 Amplicon‐based next‐generation sequencing of RSV G and F genes

Viral RNA was extracted from 200 µL aliquots of RSV‐positive nasopharyngeal specimens using the NUCLISENS^®^ easyMAG instruments (bioMerieux) according to the manufacturer's instructions. The RNA was eluted in 50 µL of elution buffer. Reverse transcription polymerase chain reaction (RT‐PCR) was carried out using the SuperScript III One‐Step RT‐PCR System (Invitrogen); the cDNAs containing the G and F genes were sequenced on the Illumina MiSeq instrument as described previously.^18^

### 2.5 Next‐generation sequence assembly

Assembly of the sequencing reads into target amplicon sequences (contigs) was performed with the Next‐Generation Sequencing Microbial Surveillance Toolbox (NGS‐MSTB)^20^ (manuscript is under review), a fully automated distributed pipeline that was implemented at AstraZeneca with a Common Workflow Language (CWL), and with a user interface based on the Galaxy bioinformatics workbench.^21^

### 2.6 Amino acid sequence analysis of the RSV F proteins

The F gene sequences in FASTA format were translated to amino acid sequences and aligned against the reference sequences derived from Netherlands RSVA/13‐005275 (GenBank accession no. KX858757) and Netherlands RSVB/13‐001273 (GenBank accession no. KX858756). Amino acid variation was determined and reported from pairwise alignments of sample sequences to the reference. The mapping of antigenic site changes onto the pre‐fusion and post‐fusion forms of the F protein structure has been described previously.^18^

### 2.7 Subtyping and genotyping analysis based on the RSV G gene

The assignment of RSV genotypes was performed with a combination of a nearest neighbor classifier and phylogenetic clustering, using a reference database of the previously described genotypes.^22^ Phylogenetic analyses were conducted in mega7.^23^ RSV G gene sequences were translated into protein sequences and aligned using Muscle^24^ along with the reference sequences (GenBank accession no. KX858754 and KX858755) for RSV AG and BG, respectively. Phylogenetic trees were generated using the maximum likelihood method based on the JTT matrix‐based model and were visualized and annotated using ITOL v3.^25^

## 3 RESULTS

### 3.1 Distribution of RSV subtypes and genotypes in South Africa

A total of 742 RSV‐positive nasopharyngeal samples were collected from HIV‐unexposed (HU, n = 544, 73.3%) and HIV‐exposed‐uninfected (HEU, n = 198, 26.7%) hospitalized infants in South Africa between 2015 and 2017. From this sample pool, 147 samples (50 samples in 2015; 49 samples in 2016; 48 samples in 2017) were successfully PCR‐amplified and sequenced (Figure 1A). Overall, more samples had RSV A in 2015 (58.0%) and 2016 (61.2%) while RSV B was more prevalent in 2017 (68.8%) (Table 1). The seasonal and HIV exposure selection yielded a group of samples mostly from patients 1‐6 months of age with more males than females (ratios 1.6, 1.2 and 1.2 in 2015, 2016, and 2017, respectively). Most infant patients were hospitalized for more than 24 hours (62.0% in 2015, 67.3% in 2016, and 66.7% in 2017). There was no significant correlation between length of stay and HIV exposure status. Slightly more RSV A cases than RSV B cases were sequenced from HEU infants in 2015 and 2016. However, due to the small sample size, there was no statistically significant difference in the RSV A and B subtype distribution between HU and HEU infants in the selected samples.

**Table 1:** Demographics by RSV A or B infection in South African infants during 2015‐17

	Total	2015			2016			2017		
	(%)	Total	RSV A	RSV B	Total	RSV A	RSV B	Total	RSV A	RSV B
Number										
(%)	147	50	29 (58.0%)	21 (42.0%)	49	30 (61.2%)	19 (38.8%)	48	15 (31.3%)	33 (68.8%)
Age group										
≤1 mo	11%	9	6 (66.7%)	3 (33.3%)	5	3 (60.0%)	2 (40.0%)	3	2 (66.7%)	1 (33.3%)
>1 to ≤3 mo	32%	16	9 (56.3%)	7 (43.8%)	16	10 (62.5%)	6 (37.5%)	15	4 (26.7%)	11 (73.3%)
>3 to ≤6 mo	39%	17	8 (47.1%)	9 (52.9%)	15	9 (60.0%)	6 (40.0%)	25	9 (36.0%)	16 (64.0%)
>6 to <12 mo	18%	8	6 (75.0%)	2 (25.0%)	13	8 (61.5%)	5 (38.5%)	5	0 (0.0%)	5 (100.0%)
Gender										
Male	57%	31	18 (58.1%)	13 (41.9%)	27	19 (70.4%)	8 (29.6%)	26	10 (38.5%)	16 (61.5%)
Female	43%	19	11 (57.9%)	8 (42.1%)	22	11 (50.0%)	11 (50.0%)	22	5 (22.7%)	17 (77.3%)
Length of stay†										
≤24 h	35%	19	9 (47.4%)	10 (52.6%)	16	10 (62.5%)	6 (37.5%)	16	5 (31.3%)	11 (68.8%)
>24 h	65%	31	20 (64.5%)	11 (35.5%)	33	20 (60.6%)	13 (39.4%)	32	10 (31.3%)	22 (68.8%)
HIV exposure status										
HEU	39%	21	15 (71.4%)	6 (28.6%)	19	13 (68.4%)	6 (31.6%)	17	5 (29.4%)	12 (70.6%)
HU	61%	29	14 (48.3%)	15 (51.7%)	30	17 (56.7%)	13 (43.3%)	31	10 (32.3%)	21 (67.7%)

^†^ No significant correlation between length of stay and HIV exposure status.

The distribution of the RSV A and B genotypes based on the G protein sequence analysis, and by month of year when identified, is shown in Figure 1B. RSV A was most prevalent in 2015 (58.0%) and 2016 (61.2%), while RSV B was most prevalent in 2017 (68.8%). Among RSV A isolates, ON1 genotype was the most prevalent from 2015‐2017 (82.8%, 93.3%, and 86.7%, respectively) with the remainder being NA1. For RSV B, the BA9 genotype gradually replaced BA10 as the dominant genotype from 2015 to 2017 (14.3%, 63.2%, and 97.0%, respectively).

**Figure 2:**
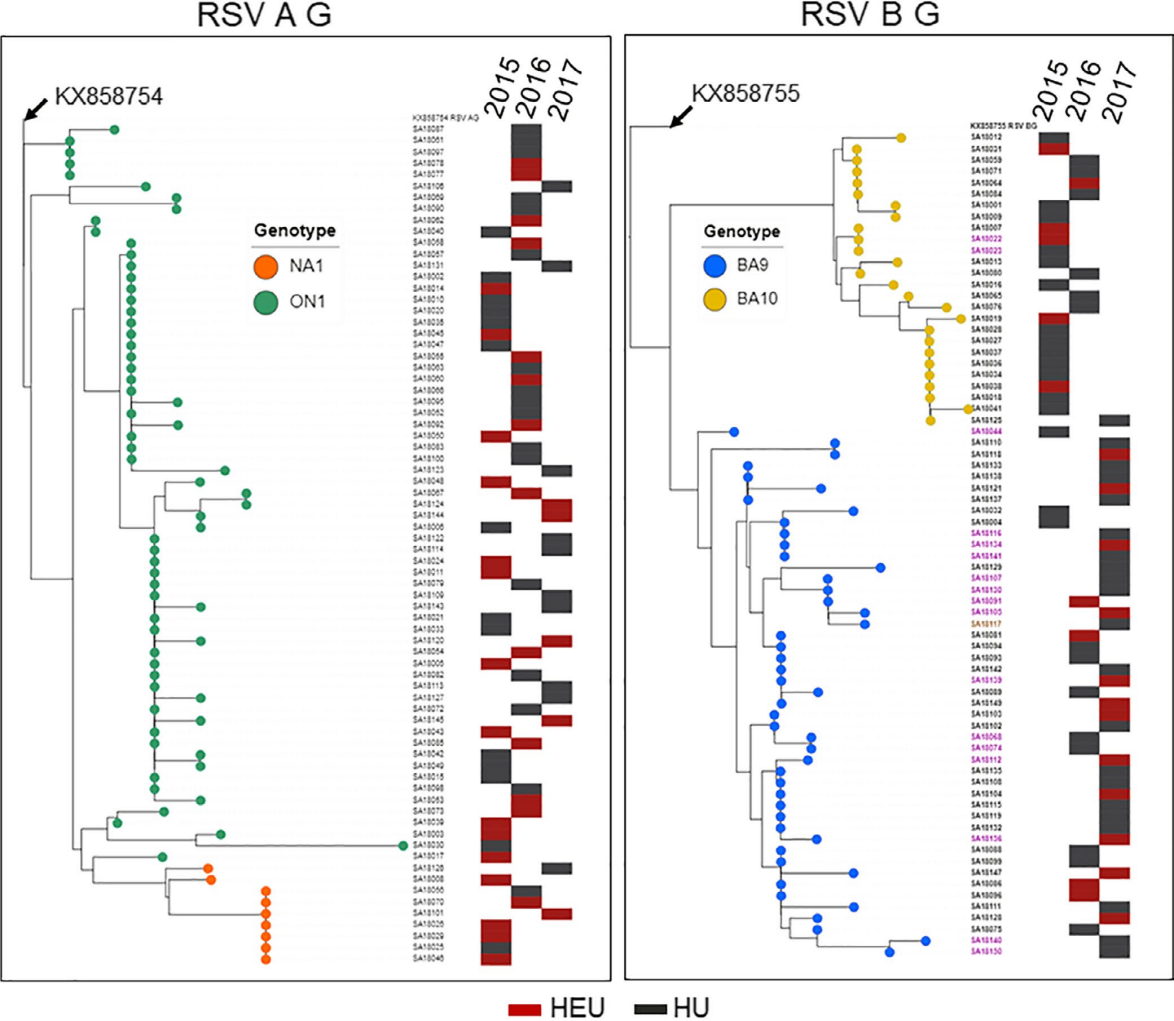
Phylogenetic trees based on the second hypervariable region of the G protein. The reference sequences KX858754 and KX858755 were used to root RSV A G and B G trees, respectively. The evolutionary history was inferred by using the maximum likelihood method based on the JTT matrix‐based model in mega7. Phylogenetic trees were visualized and annotated using ITOL v3. Genotypes are indicated by leaf color (RSVA: NA1 orange, ON1 green, RSV B: BA9 blue, BA10 yellow). Year of sample collection and the HIV exposure status (HEU red, HU black) are indicated by the presence of a filled bar in the respective year column. RSV B: sample names in purple indicate G protein extension, brown indicates truncation

The RSV A and B phylogenetic trees based on the G‐HVR2 amino acid sequences are shown in Figure 2. RSV A ON1 (65/74, 87.8%) and NA1 (9/74, 12.2%) sequences formed distinct branches, with NA1 at the base of the tree, mostly from 2015. Similarly, RSV B BA9 (47/73, 64.4%) and BA10 (26/73, 35.6%) also formed distinct branches with BA10 at the top of the tree, also primarily from 2015. There were no obvious RSV clusters for HU nor HEU samples.

G protein length polymorphisms were observed in a number of RSV B strains (isolates in purple and brown in Figure 2). Approximately 23.3% (17/73) of RSV B samples had a mutation in the stop codon from TAA to CAA (Q) resulting in 7 amino acid extension of the G protein. This polymorphism in the carboxyl terminus of the G protein has also been observed in RSV isolates from the USA.^18^ The 7 amino acid extension was more frequently found in BA9 (15/47, 31.9%) than BA10 (2/26, 7.7%). In addition, there was one BA9 isolate with a premature stop codon resulting in a 21 amino acid truncation of the G protein.

### 3.2 Polymorphisms in the RSV F protein

**Figure 3:**
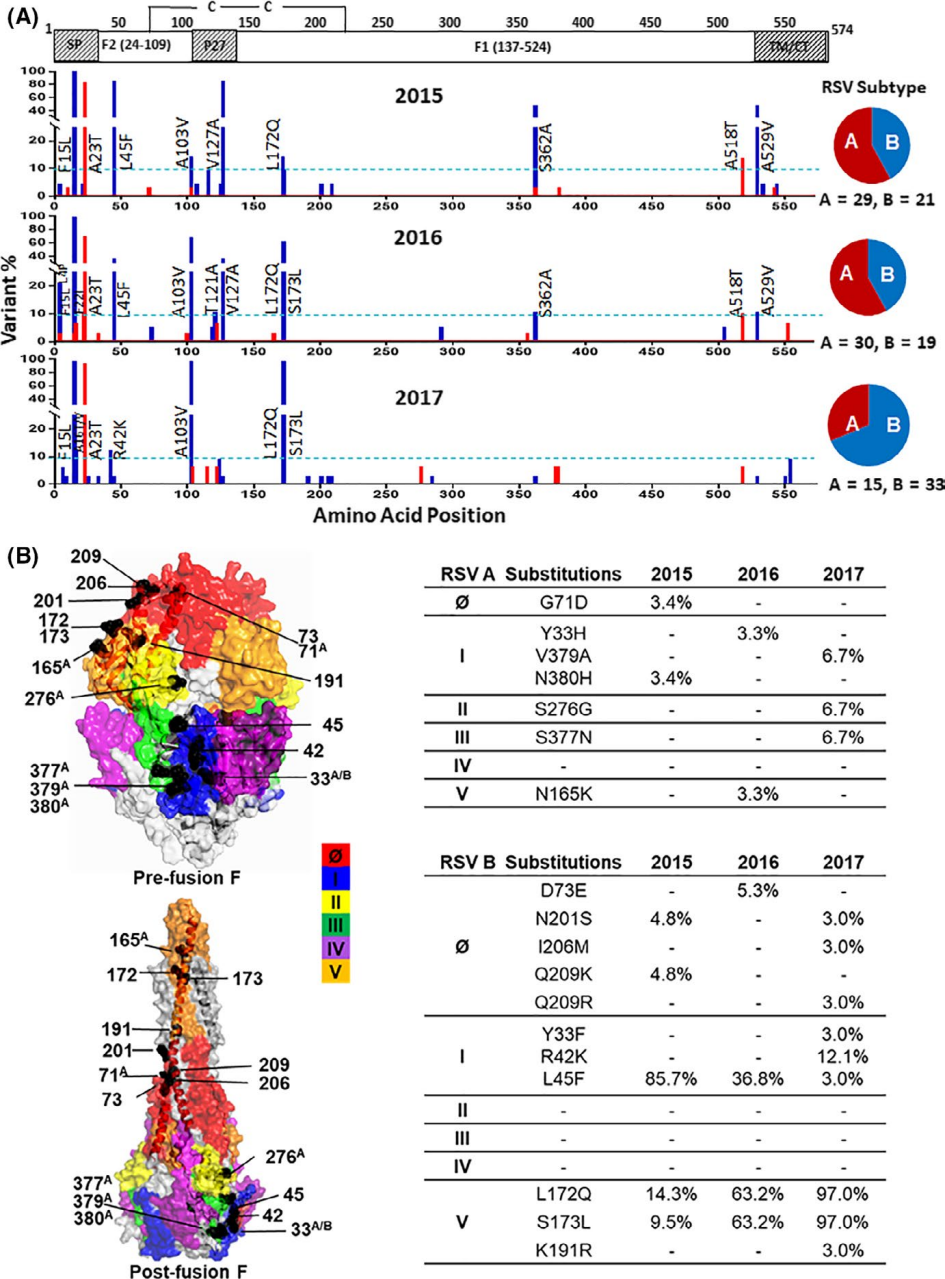
Polymorphisms in the RSV A and B Fusion proteins. A, Plots of amino acid variation frequency by position of F protein open reading frame (1‐574) compared to the KX858757 (RSV A F) and KX858756 (RSV B F) reference sequences based on year. SP, signal peptide; P27, cleaved peptide of 27 AA residues, TM, transmembrane domain, CT, cytoplasmic tail. Red, RSV A; Blue, RSV B. Variations at a frequency >10% (dashed lines) in each season relative to the references are labeled. B, Polymorphisms observed in the antigenic sites of RSV F protein. RSV F protein pre‐fusion and post‐fusion conformations were based on the Protein Data Bank files of 5UDE and 3RRR, respectively. Antigenic sites (Ø, I, II, III, IV, and V) are colored. Red: site Ø, blue: I, yellow: II, green: III, purple: IV, orange: V. Amino acid variations in these antigenic sites and their frequency for each season are listed in the tables on the right. ^A^Indicates variation only showed in RSV A. ^A/B^Indicates residues that showed variations in both RSV A and RSV B. Other numbers indicate variations in RSV B only

The F protein sequences derived from the 147 isolates were analyzed by comparison to the 2013 reference sequences RSV A/13‐005275 and RSV B/13‐001273. Of 74 RSV A F protein sequences, 6 (8.1%) were identical to the RSV A reference sequence, and 37 (50.0%) were nearly identical to the reference except for an A23T change in the signal peptide. None of the 73 RSV B F protein sequences was identical to the RSV B reference sequence. However, 29 (39.7%) RSV B F sequences were identical to the sequence derived from the predominant RSV B isolate circulating in the USA during the 2015‐2017 seasons, containing F15L/A103V/L172Q/S173L substitutions compared to the reference strain.^18^ The frequency and individual polymorphisms in the RSV A and B F protein sequences are presented in Figure 3A.

Overall, the RSV A F protein had fewer amino acid sequence changes at a frequency >10% compared to RSV B. There were only 2 amino acid changes in the RSV A F protein detected in more than 10% of the isolates, the A23T in the signal peptide, and an A518T near the transmembrane domain. RSV B F protein displayed several amino acid differences at a frequency >10% compared to the reference strains, and additional changes occurred over the 3‐year period. While most changes were detected continuously from 2015 to 2017 (F15L, A103V, L172Q, and S173L), some of these observed in the 2015 isolates were lost by 2017 (L45F, V127A, S362A, and A529V).

The polymorphic amino acid changes in the six major antigenic sites (Ø, I‐V)^9^, ^10^, ^26^ of the F protein were further examined, and amino acid variations at these sites with frequency >1% in their respective seasons were labeled on the pre‐fusion and post‐fusion forms of the protein structure (Figure 3B). There were a total of 7 amino acid differences in the F protein antigenic sites of RSV A with frequencies ranging from 3.3% to 6.7% and 11 amino acid differences in the F protein of RSV B with frequencies ranging from 3.0% to 97.0%. Some differences were only observed in a specific season and were not detected in the subsequent season, such as RSV A G71D at site Ø, Y33H and N380H in site I, N165K in site V, and RSV B D73E and Q209K at site Ø. However, L172Q and S173L at the antigenic site V of RSV B increased from 14.3% and 9.5% in 2015 to 97.0% by 2017.

### 3.3 Sequence comparison of RSV F proteins from South Africa and the USA

**Figure 4:**
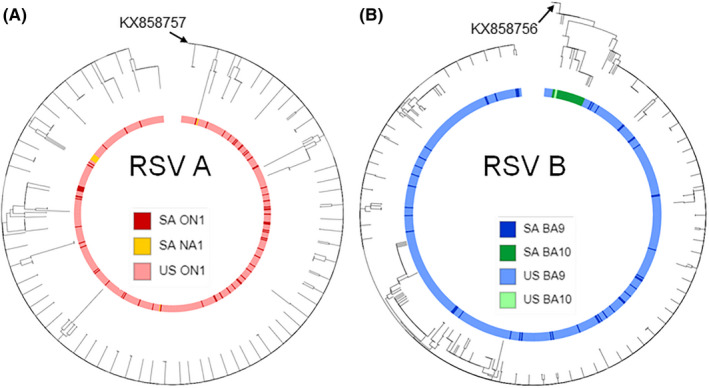
Phylogenetic analysis of RSV A and B Fusion proteins from South Africa and USA (2015‐17). Phylogenetic trees were generated by using the maximum likelihood method based on the JTT matrix‐based model in mega7. Trees were visualized and annotated using ITOL v3. Genotypes are indicated by color ring

RSV F protein sequences from South Africa were compared to those collected in the USA OUTSMART‐RSV study during the same period despite their age difference between these two populations.^18^, ^27^ All the RSV A isolates from the USA were ON1, and all but two RSV B (BA10) USA isolates were BA9 (Figure 4). Most of the South Africa RSV A F proteins share high sequence homology with the RSV A F proteins from the USA. Some South Africa ON1 genotypes clustered together in the F phylogenetic tree close to the NA1 genotype. Most RSV B BA9 isolates had similar sequence clustering compared to the USA OUTSMART‐RSV isolates. However, the RSV B F protein sequences from the South African BA10 genotypes clustered together with at least one amino acid difference from the USA BA10 strain, suggesting a local circulation pattern.

## 4 DISCUSSION

The South Africa RSV cohort samples studied here represent local RSV epidemiology in the 2015‐2017 period. We showed that the HIV exposure status of these infants did not affect the distribution of RSV subtype or genotype. We also noted a greater proportion of RSV‐positive samples in male infants, compared to those of females, which is consistent with the findings from the USA surveillance study^18^ and a recently reported study in Spain.^28^

The RSV A ON1 genotype characterized by a 72 nucleotide (23 amino acid) duplication in the G‐HVR2 has replaced NA1 and other genotypes as the most prevalent strain in the world over the past 10 years.^18^, ^29^ The RSV B BA9 genotype, which replaced BA10 over the three study years in South Africa (14.3%, 63.2%, and 97.0%, respectively), has become the most prevalent RSV B genotype worldwide.^30^ Both BA9 and BA10 genotypes are characterized by a 60 nucleotide (20 amino acid) duplication in the G‐HVR2 region.^31^ The duplication sequence has been hypothesized to increase viral fitness.^32^ In addition, we also observed a seven amino acid extension of RSV B G protein in 17 (23%) isolates. Interestingly, this same seven amino acid extension was also found in RSV B isolates from the USA surveillance study conducted during the same period, and at a similar frequency of 22%.^18^ The biological significance of the seven amino acid extension observed for RSV B strains remains to be determined. The RSV A NA1 and RSV B BA10 genotypes were mostly absent in the USA from 2015‐2017,^18^ but they were still present in South Africa, although at decreasing frequencies. The RSV peak season in South Africa (March to May during the 2015‐17 RSV seasons) is different from the Northern hemisphere, where the RSV epidemic peaks between November and March. Our data suggest that RSV sequence evolution in South Africa might be lagging behind the RSV evolution in the USA.

RSV epidemiology studies in South Africa prior to 2012 have been reported,^33^, ^34^ showing that positive selection drove RSV A and B evolution and replacement of genotypes. However, these studies were limited to the G protein gene sequencing only. Since F protein is a major target for anti‐RSV monoclonal antibodies, it is critical to understand F protein sequence evolution and antigenicity changes in contemporary circulating strains. Based on analysis of the antigenic sites in the RSV F protein, RSV B had more variability than RSV A (Figures 3 and 4). The RSV A F protein had 7 changes at 5 antigenic sites at a frequency <10% compared with the reference strain. The RSV B F protein had a total of 11 changes distributed over 3 antigenic sites, with 2 changes in antigenic site I and 2 changes in antigenic site V at a frequency >10%. Thus, although the F protein structure is generally conserved, changes at various antigenic sites can occur.

The sequence containing L172Q/S173L changes in the antigenic site V (target of suptavumab^13^, ^14^) increased from <15% in 2015 to 97.0% by 2017, which was also observed in the USA and other countries over the same period.^18^, ^35^ These changes led to the resistance of RSV B to suptavumab.^36^ The I206M/Q209R changes at antigenic site Ø (target of nirsevimab) were detected at a low frequency of 3.0% in 2017, but were present in the USA RSV isolates at a frequency of approximately 19% in the 2016/17 RSV season.^18^ Importantly, these two amino acid changes do not affect the susceptibility of RSV neutralization by nirsevimab.^37^

One limitation of this study is the selection of 150 samples out of 742 samples collected, and there is a possibility that the data may have a selection bias. The comparison of the SA RSV isolates with the USA data was based on the F protein sequence that showed some similar RSV sequence patterns between the two countries. The comparison of the G protein sequences between the SA RSV isolates and the USA data was not performed due to incomplete protein sequences of some USA samples from the 2015‐16 season. Based on the comparison of the available G nucleotide sequences between the two countries, some SA RSV isolates clustered together whereas the rest of the SA isolates showed similar sequence patterns to the USA data in the phylogenetic trees for both RSV A and B strains. Our data confirm previous observations that emergence of a new RSV strain in one area could circulate globally and contributes to the recurrence of annual epidemics. The innovative sequencing data analysis tools developed in this study provided a high‐throughput solution for sequencing and analyzing a very large number of samples in a short period of time.

The global characterization of the RSV genomic sequences is necessary for anti‐RSV therapeutics and vaccine development. Surveillance studies can detect and monitor emerging strains for their susceptibility to antivirals and anti‐pathogen monoclonal antibodies, and antigenic changes that may affect vaccine effectiveness. Continued global surveillance of RSV is important in order to understand the spread and evolution of RSV, and help predict the outcomes of prophylactic and therapeutic interventions.

## CONFLICT OF INTEREST

HL, BL, DET, AT, DW, HJ, and MTE are employees and shareholders of AstraZeneca when the study was conducted; SAM, NS and MCN had no competing interests.

## AUTHOR CONTRIBUTIONS

Conceptualization: MCN, MTE, HJ, SAM and NS; Methodology: HL, BL, DET, AT; Data analysis: HL, BL, DET, AT, DW, HJ, MTE; Writing Manuscript: HL, BL, HJ, MTE and MCN; Review and Approval: HJ, MTE, MCN, SAM, DW, DET, AT.
